# Alkyl-capped copper oxide nanospheres and nanoprolates for sustainability: water treatment and improved lubricating performance

**DOI:** 10.1080/14686996.2019.1621683

**Published:** 2019-06-26

**Authors:** Christian Chimeno-Trinchet, Alfonso Fernández-González, Josefa Ángela García Calzón, Marta Elena Díaz-García, Rosana Badía Laíño

**Affiliations:** Faculty of Chemistry, Department of Physical and Analytical Chemistry, University of Oviedo, Oviedo, Spain

**Keywords:** CuO nanoparticles, superficial functionalization, oil medium, tribological properties, lubricant additive, dye-remover additive, 60 New topics / Others: Nanoparticles, 301 Chemical syntheses / processing, 500 Characterization, 503 TEM, STEM, SEM, 200 Applications

## Abstract

Metal oxide nanoparticles of different nature have been used in different fields such as therapeutics, biomarkers, tribology or environmental remediation, among others. Besides, the surface modification of such nanoparticles is of particular interest to bring designed functions. In this paper we describe the synthesis of CuO nanoparticles with two different geometries (spherical and prolate) and decorated with long alkyl chains in order to use as dye removers by adsorption and/or photo-degradation of a persistent model dye (Congo Red) and as lubricant additives to improve the tribological performance of base lubricant oils. Alkyl-functionalized CuO nanoparticles demonstrated a high stability in oily suspensions and an improvement in the friction reducing the CoF ca. 26%; the alkyl-decorated nanoparticles showed also higher adsorption kinetics for Congo Red than the neat ones following a pseudo-second-order trend, although with lower adsorption efficiency. The synthesis, surface modification and physic-chemical characterization of spherical and prolate CuO nanoparticles are described as well as their applications as lubricant additives and Congo Red photocatalytic removal.

## GRAPHICAL ABSTRACT

**Figure:**
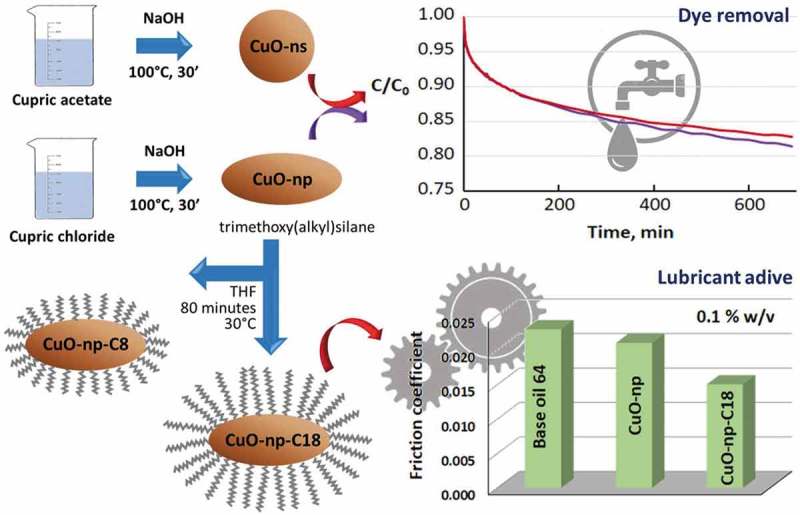

## 1. Introduction

According to the European Environment Agency (EEA, 2008), there is continuing concern about loss of biodiversity, acidification of freshwater, waste production and management, global warming, extreme weather events, urban air pollution, and environmental noise, among others. Over the last 25 years, the knowledge about the unique properties of nanomaterials has rapidly evolved and engineered novel nanomaterials are currently designed to address social challenges that affect from environmental research to energy efficiency. In this context, environmental demands include dye removal from water. Dyes from different industries (paper and pulp, leather, cosmetics, food industries and textile) are environmental pollutants that enter the environment through different sources. Besides, the textile industry uses not only dyes but also a large variety of chemicals (detergents, oils, latex and glues, softeners, wetting agents and other special chemicals) that can be present in processing effluents, and it is considered as the most polluting of all the industrial sectors. In particular, the loss of dyes in effluents from textile industry may reach up to 140,000 tons every year during dyeing and finishing processes [1]. Aside from the undesirable color that very small amounts of dyes (<1 ppm for some dyes) impart to water, dissolved dyes may also inhibit sunlight penetration in water and/or may be toxic, thus affecting aquatic life. Several procedures for removing dyes from water have been proposed, being the most common adsorption [2], flocculation/flotation processes or degradation [3]. Regarding the last process, nanomaterials have proved to be an interesting research field for dye removal.

One of the objectives of this paper is to explore the feasibility of using CuO nanoparticles (CuO-NPs) with different geometry (nanospheres and nanoprolates) to remove undesirable dyes in water by adsorption and/or by enhanced photochemical degradation. Congo Red, used as an example of persistent dye in water, is toxic and possibly carcinogenic and mutagenic to many organisms, even for human.

On the other hand, development of new lubricants based on the use of nanomaterials for improved machine performance, which results in reduced energy losses caused by some tribological issues and/or preventing component failure, is another key contribution to improve efficiency in many industrial processes [4,5]. For example, in a wind energy system (wind turbine), friction within the gearbox during the conversion of wind kinetic energy into mechanical or electrical energy, is one of the important sources of energy losses in these systems. To increase the wind turbine efficiency, different nanoparticle-based lubricants have been used [6,7] Although long-term stability of such nanolubricant suspensions is a crucial prerequisite for an effective lubrication performance, information about the dispersion method used, and the stability of the nanolubricants is scarce or even lacking in the related literature [8].

With the aim to develop stable and long-lasting nanoparticle base lubricants and to demonstrate their potential as dye removers, in this paper we deal with the synthesis of raw spherical and prolate shaped CuO-NPs and their surface coating with long alkyl chains. Several metal oxides nanoparticles have been used as friction modifiers showing four main behaviors: tribofilm formation, roll between surfaces acting as ‘nano ball bearings’, mending worn surfaces due to nano-size and polishing [9]. Among those metal oxides, CuO-NPs have been previously used as a lubricant additive, showing promising results such as improving wear resistance and reducing friction [10,11]. In this work we addressed the engineering of CuO-NPs of different morphology by modification of their surface using long alkyl chains, C_8_ and C_18_, providing them with a hydrophobic character. The guide of the design has been the final applications of such modified CuO-NPs. Firstly, use the modified CuO-NPs for persistent Congo Red removal from water by adsorption and/or by a photocatalytic way. Also, a second objective is the use as nanoparticle additive in base oil (with no other additive) to improve the tribological performance of the lubricant. The CuO-NPs developed to tackle both objectives were fully characterized by electron microscopy in order to study the shape and size distribution of the nanoparticles. Experimental results showed that alkyl-functional CuO-NPs were highly stable in oily suspensions compared to the naked ones. The performance of CuO NPs as dye-removers and lipophilic CuO NPs as lubricant additives to improve tribological properties of base oils is also outlined.

## 2. Experimental details

### 2.1. Materials

Copper(II) acetate monohydrate 98%, trimethoxy(octadecyl)silane 90% and tetrahydrofurane (THF) 99.5% were purchased from Acros; NaOH 98% and Congo Red (disodium;4-amino-3-[[4-[4-[(1-amino-4-sulfonatonaphthalen-2-yl)diazenyl]phenyl]phenyl]diazenyl]naphthalene-1-sulfonate) indicator grade were bought from Alfa Aesar. Copper(II) chloride dihydrate, 99% and glacial acetic acid were acquired from WVR, whereas trimethoxy(octyl)silane 96% and ethanol HPLC grade originated from Aldrich and Millipore, respectively. Base oil 68, for tribological assays, was kindly provided by Repsol S.A.

A Congo Red standard solution 1.10^−3^ M was prepared every two days and kept at 4°C in the dark to avoid photo-degradation.

### 2.2. Instrumentation and methodology

UV-Vis experiments were performed using an Agilent Cary 60 spectrophotometer (Agilent, USA) equipped with a Peltier system for temperature control and a stirring cuvette holder. Kinetics experiments and UV-Vis spectra were taken at 15°C with constant stirring. Fourier-transform infrared (FTIR) spectra were recorded with a Varian 670-IR spectrometer (Varian, USA) and a Golden Gate attenuated total reflection (ATR) device using a diamond crystal. Spectra were recorded between 4000 cm^−1^ and 600 cm^−1^, with 16 scans at 4 cm^−1^ resolution. Samples were measured in the ATR device without further processing. Solid samples were pressed with an anvil while liquid samples (suspensions and solutions) were poured on the diamond’s surface and then evaporated under N_2_ current.

X-ray diffraction (XRD) patterns were recorded with a PANalytical X’Pert Pro setup (PANalytical, UK) using Cu Kα radiation (λ = 1.5418 Å) and a Ni filter.

Thermogravimetric measurements were carried out using a Mettler Toledo thermogravimetric analysis/synchronous differential thermal analysis (TGA/SDTA) (Mettler Toledo, USA) 851 instrument in pure N_2_ atmosphere. Thermograms were obtained at a heating rate of 10°C min^−1^ from 25°C to 800°C under nitrogen atmosphere flowing at 10 mL·min^−1^. For thermogravimetric assays sample weight ranged from 65 to 85 mg, depending on the sample analyzed.

Transmission electron microscopy (TEM) images were recorded with a JEOL-2000 EX-II microscope (JEOL, Japan) operated at 160 kV or with a JEOL-JEM 2100 F (JEOL, Japan)  at 200 kV. TEM experiments were prepared by suspending a few milligrams of dry powder of nanoparticles into 1.5 mL ethanol. Aliquots of such suspension were dropped onto a carbon-coated copper grid and dried for inspection in the microscope. The mean particle size was analyzed from the digitized images with ImageJ Tool software where at least 150 observations were performed for every dimension.

X-ray photoelectron spectroscopy (XPS) experiments were performed in a SPECS (Germany) spectrometer using a monochromatic Al K_α_ radiation (1486.74 eV) working in Fixed Transmission mode with Large Area for electromagnetic lenses. High-resolution spectra were taken using 0.1 eV step energy and 30 eV as pass energy, with a different number of scans according to the relative concentration of the elements. Survey spectrum was taken with 1 eV in energy step and 90 eV in pass energy.

Photoluminescence spectra were acquired using a Varian Cary Eclipse fluorimeter (Varian, USA) using 5 nm slits in both excitation and emission monochromators, with a wavelength scan speed of 1200 nm·s^1^.

Wear assays were carried out in a Bruker UMT3 tribometer (Bruker, USA), applying a normal load via a closed-loop servomechanism. Load and friction force were determined through strain-gages. Every test-section component was cleaned in heptane and ultrasounds for 3 min and then rinsed with ethanol. Finally, the components were dried under hot air before and after the test. The worn surfaces were studied with a Leica 6 DCM3D confocal and interferometric microscope (Leica, Germany), enabling the quantification of wear loss.

A Bandelin Sonopuls HD2200 (Bandelin, Germany) coupled with a Titanium probe 13 mm Zetasizer was used for ultrasonication.

Selecta Ultrasons HD (Selecta, Switzerland), ultrasound bath, was used at 120 W and 40 kHz at room temperature.

### 2.3. Synthesis of CuO nanoparticles

**CuO nanospheres** were synthesized using the procedure described by Cetinkaya et al. [12] with slight modifications. Nanoparticles were obtained from cupric acetate, prepared by mixing 300 mL of 0.02 M cupric acetate with 1 mL of glacial acetic acid to prevent hydrolysis, diluting the mixture to 300 mL with distilled water. Then, the solution was heated at 100°C under reflux. After 30 min, 0.8 g NaOH was dissolved in the minimum possible amount of water and added to the hot solution. After the reaction, a black precipitated of CuO nanospheres was obtained, being the final pH of the solution 7.0.

**CuO nanospheroids** (nano-prolates) were obtained adapting the procedure described by Krishnan [13], using cupric chloride as initial salt. Briefly, 300 mL of a 0.04 initial solution of cupric chloride was mixed with a concentrated NaOH solution obtained by dissolving 1 g NaOH in the minimum amount of water. The final cupric–NaOH mixture was left to react for 30 min at 100°C under reflux.

In both instances, the final nanoparticles were separated by centrifugation during 5 min at 5000 rpm. The precipitates were purified by re-suspension in water (x1), centrifuged and then re-suspended in ethanol (x2) and centrifuged for 10 min. Finally, the purified nanoparticle pellets were dried in oven at 80°C, and final products were labeled as CuO-ns (CuO-nanospheres) and CuO-np (CuO-nanoprolates).

#### 2.3.1. Surface modification of nanoparticles

Both, CuO-ns and CuO-np, were surface modified using silane coupling agents (structures in Figure S1, Supplementary Information). The coating process was carried out according to the method described by Sadollahkhani et al. [14] and Malwal and Gopinath [15], with minor modifications. 1.5 g of the selected nanoparticles was dispersed in 30 mL of THF using an ultrasound bath for 30 min. Then, 3 mL of trimethoxy(octyl)silane were added to the suspension, and the final mixture was left to react for 80 min at 30°C. The final product was separated by centrifugation (5 min, 5000 rpm) and then washed twice with THF and once with ethanol so as to remove the excess of reactants. The octyl-modified nanoparticles (labeled as CuO-ns-C_8_ and CuO-np-C_8_) were dried in a vacuum line at room temperature for 24 h. In order to yield more hydrophobic nanoparticles, CuO-ns, and CuO-np surface modification was also performed using 5 mL of trimethoxy(octadecyl)silane (other reagents and procedures as described above). Final nanoparticles were labeled as CuO-ns-C_18_ and CuO-np-C_18_, respectively.

#### 2.3.2. Wear tests

A selected amount of raw or surface modified nanoparticles were mixed with 20 g of base oil 68 and sonicated at 40% amplitude in a water bath for 20 min. Tribological properties were evaluated by a ball-on-plate test using 9.5 mm diameter AISI 52,100 chrome steel balls of hardness 63 HRC (hard Rockwell scale) and surface finish <0.05 microns Ra (roughness average) against a softer AISI 52,100 steel disk of hardness ranging from 190 to 210 HV (Vickers hardness) and surface finish below 0.02 microns Ra). Assays were carried out using 4.5 mL of nanoparticles suspensions as a lubricant for 20 min, at room temperature, at 4 mm amplitude, 15 Hz of reciprocating amplitude and a normal load of 60 N. Each test was conducted three times, with the friction force and normal load monitored online. Volume loss, due to wear, was evaluated after the tribological analysis with a confocal microscope.

#### 2.3.3. Degradation and adsorption tests

The degradation studies were carried out on 2.5 10^−4^ M of Congo Red solutions containing a selected amount of pristine or surface treated nanoparticles. The activity of all tested nanoparticles was followed spectrophotometrically at 498 nm, the wavelength of maximum absorption for Congo Red. Solutions were centrifuged to obtain a clear nanoparticle-free solution before measurement.

Adsorption kinetic studies were performed in the darkness. The absorbance of such solution was continuously monitored at 498 nm in a spectrophotometric cell with stirring.

Dye degradation studies in the presence of raw and surface decorated nanoparticles were performed. Suspensions of nanoparticles in 2.5 10^−4^ M Congo Red solution were put in an air-tight flask and then exposed to either sun radiation with day & night cycles or to UV lamp with continuous illumination for five days. Zero-time spectrophotometric measurements were performed on the dye solution prior to the addition of nanoparticles. The end-time spectrophotometric measurements were taken in clear nanoparticle-free solutions obtained by centrifugation of dye-nanoparticle suspensions using a three centrifuge cycles at 5000 rpm for 5 min.

## 3. Results and discussion

### 3.1. Structural analysis

Taking into account that morphology of CuO-NPs is ruled by several experimental parameters, among which the chemical nature of the starting copper salts is an important one [16], CuO-NPs were obtained by chemical precipitation synthesis of two precursor salts: a) cupric acetate and a glacial acetic acid-water mixture using NaOH as reducing agent at 100°C and b) cupric chloride and concentrated NaOH as reducing agent at 100°C.

**Figure 1.**
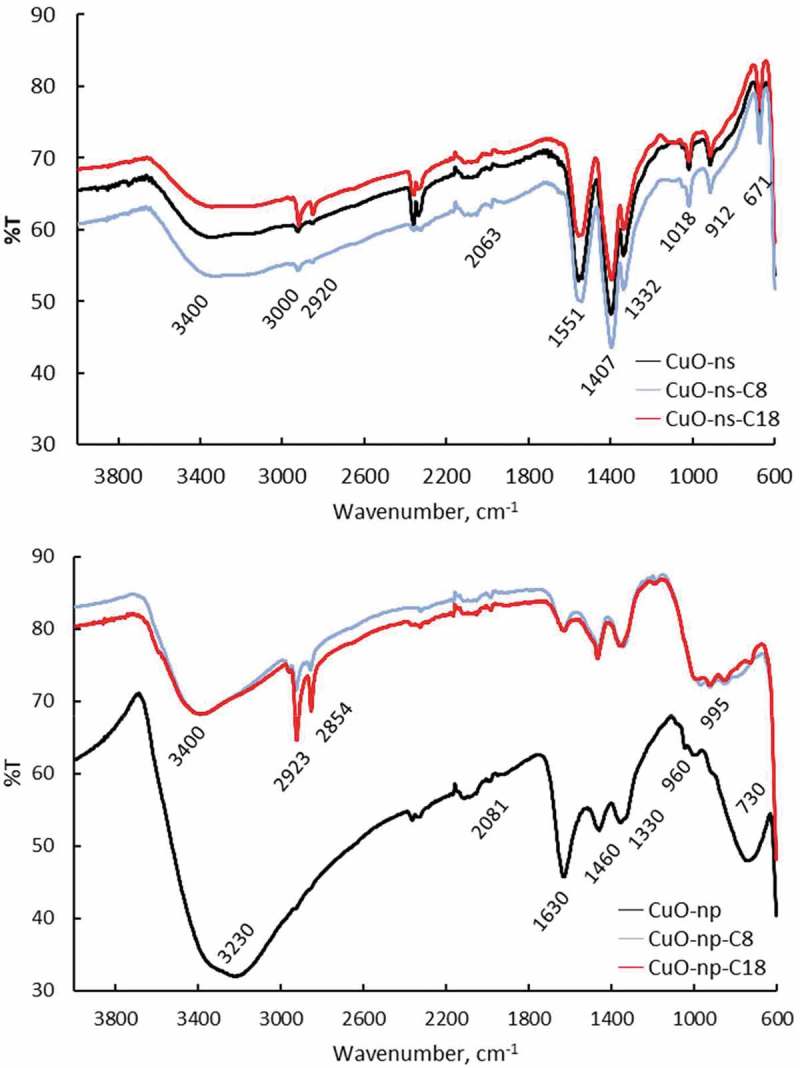
ATR-FTIR spectra of raw and functional CuO-ns and CuO-np.

Figure 1 shows the ATR-FTIR spectra of raw CuO-ns and CuO-np and the corresponding decorated ones (CuO-ns-C_8_, CuO-ns-C_18_, CuO-np-C_8_ and CuO-np-C_18_). FTIR data showed that functional CuO-ns and functional CuO-np exhibited a similar base spectral pattern to that of the uncoated material. However, the differences between the spectra of both geometries are evident. In addition to the peaks corresponding to the uncoated nanomaterial, CuO-np show additional weak peaks at 2923 cm^−1^ and 2854 cm^−1^ assigned to aliphatic C–H asymmetric and symmetric stretching [2,17], a peak at 1460 cm^−1^ arising from the C–H asymmetric deformation and a peak at 1407 cm^−1^ from symmetric deformations can be observed. In the case of CuO-np, note that the Cu-O stretching band at ~730 cm^−1^ present in the raw CuO-np shifted to ~960 cm^−1^ for the functional ones as a result of the formation of surface Cu–O–Si bonds during the derivatization process [18]. These peaks are in good agreement with those present in the alkoxysilane reagents used for functionalization (Figure S3), while peaks at 3400 cm^−1^ and 1630 cm^−1^ were assigned to the stretching and bending vibrations of the O–H group associated with adsorbed water, respectively. Although the intensity and position of these bands for CuO-ns and CuO-np were slightly different as a result of the different length of the alkyl chain, their presence in both type of nanoparticles, confirmed their successful surface capping.

The weak peaks at ~2920 cm^−1^ and ~3000 cm^−1^, which agreed with the position of CH stretching vibrations in the acetate spectrum [19] observed for raw CuO-ns could be attributed to the presence of surface acetate groups (copper acetate and glacial acetic acid used during the synthesis). Also, carboxylate bands at 1407 cm^−1^ and 1551 cm^−1^, and the medium band from COO symmetric bend at 670 cm^−1^ [20] observed for raw CuO-ns might indicate the presence of acetate groups in raw CuO-ns. However, the strong absorption band due to δ(O-H) bending vibrations of the hydroxyl groups of physically adsorbed water molecules appearing at 1640 cm^−1^ [21,22], which is clearly present in the CuO-np prepared in absence of acetate, is also present in CuO-ns overlapping with acetate bands. Upon surface modification with CuO-ns-C_8_ and CuO-ns-C_18_, peaks at ~2920 cm^−1^ and ~3000 cm^−1^ became more intense due to C-H aliphatic bonds of alkyl chains.

### 3.2. Morphology of the nanoparticles

**Figure 2.**
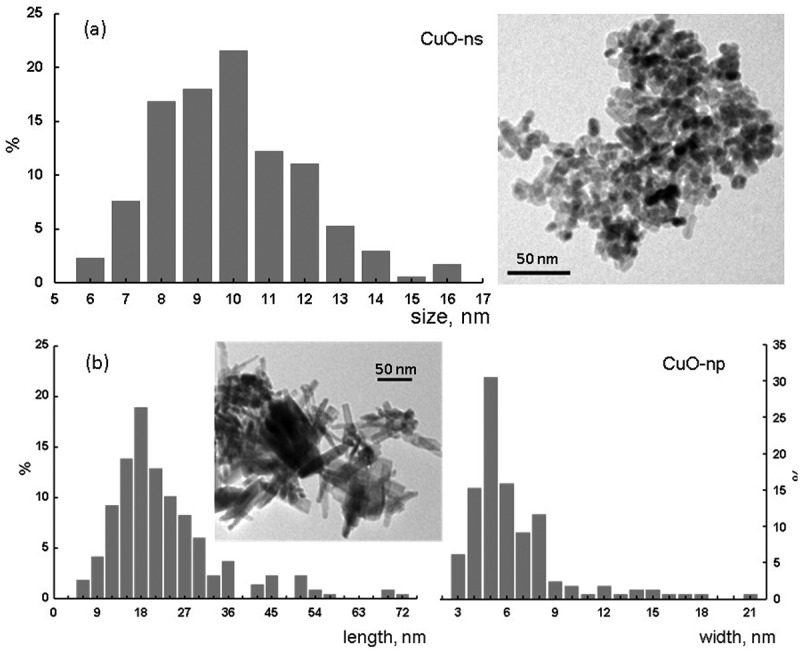
TEM images and size distributions for uncoated CuO nanoparticles prepared from a) copper acetate and b) copper chloride.

**Figure 3.**
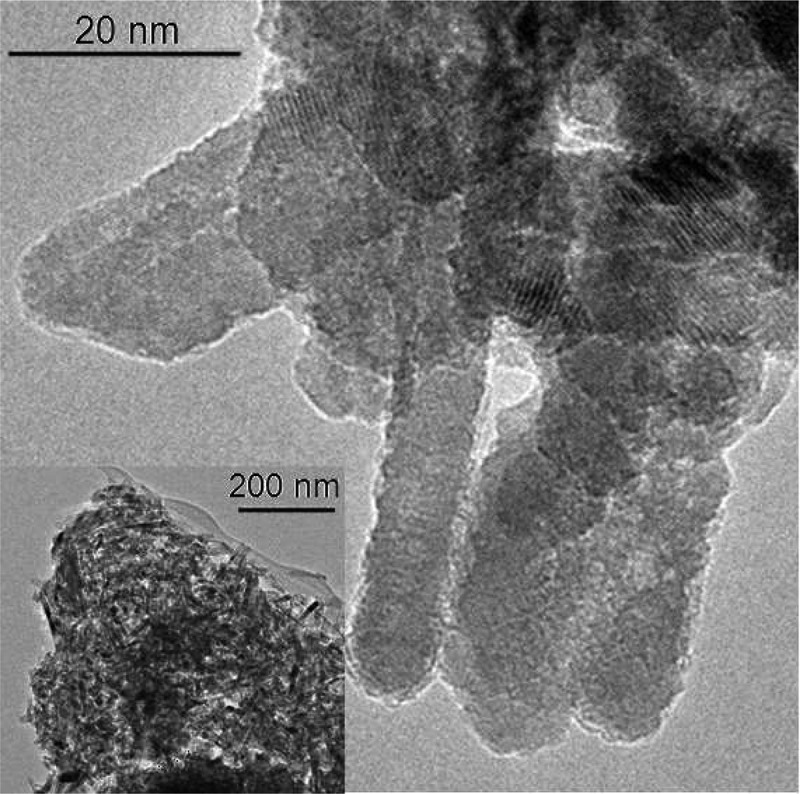
HRTEM images for CuO-np-C_18._

TEM images showed a monodisperse population of spherical CuO-ns nanoparticles (Figure 2(a)) with a mean 10 ± 2 nm diameter, while CuO-np nanoparticles exhibited needle-shape. The histograms of the CuO-np length (L) and diameter (D) are shown in Figure 2(b). Mean values of L and D, of ca. 22 and 6 nm, respectively, gave a mean aspect ratio of 3.6 which was an indicator of the impact of the copper starting material on crystal growth mechanisms and hence, on the nanoparticles morphology. Furthermore, the use of copper(II) chloride to prepare CuO-np gave rise to a moderate particle size distribution, an increase in the poly-dispersity degree, compared to the CuO-ns. No significant differences could be observed between TEM and high-resolution TEM (HRTEM) images for functional and pristine nanoparticles, whichever the precursor was used. Magnification of the HRTEM image of CuO-np-C_18_ (Figure 3) revealed that CuO showed lattice fringes, with inter-planar spacing of 2.7 Å, consistent with {110} planes and a growth along a direction parallel to {001} (dominant exposed crystal planes).

**Figure 4.**
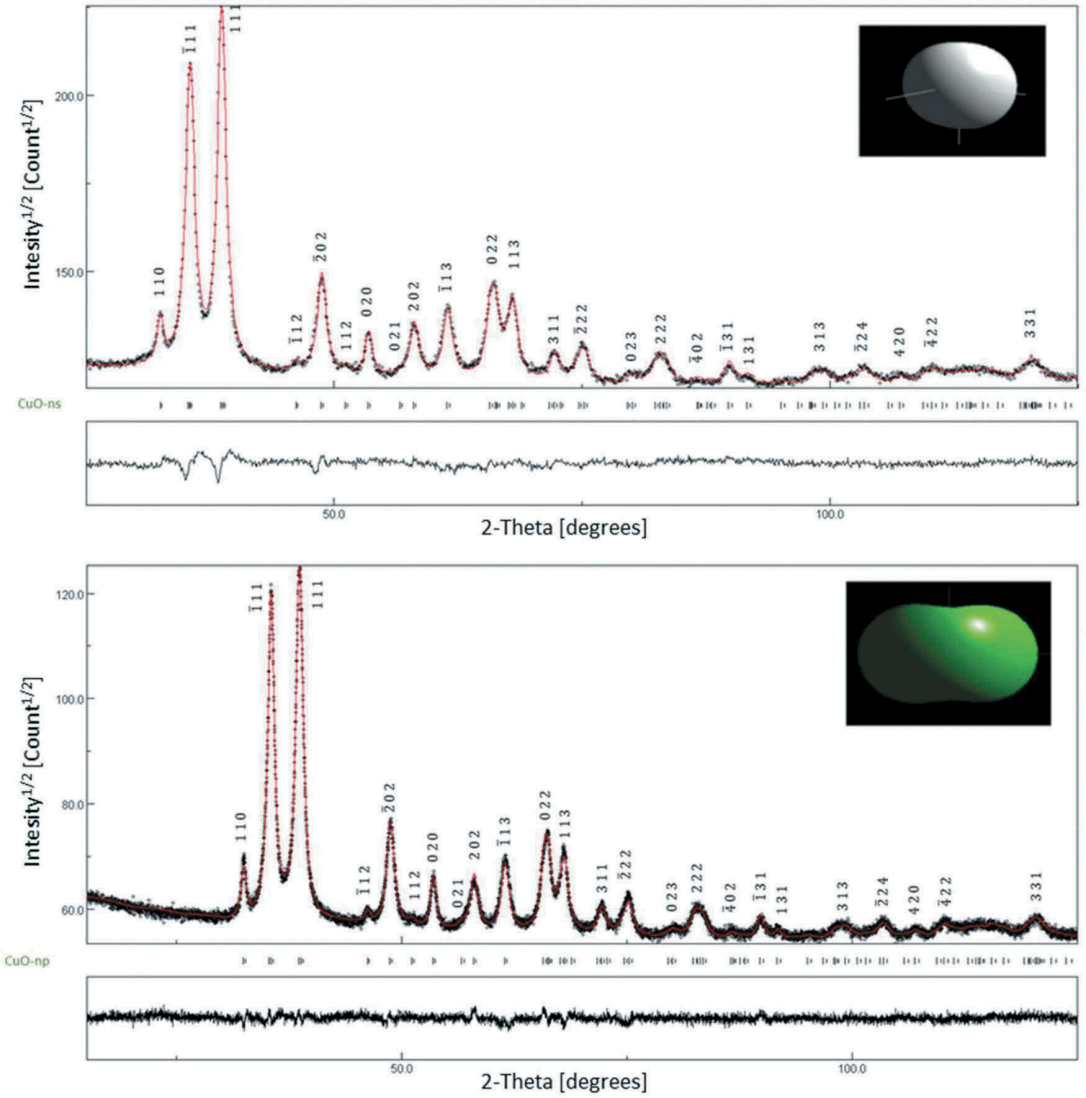
Diffractograms of CuO-ns and CuO-np samples. (◉) XRD data, (–) Rietveld refinement fit and Bragg position peaks. Insets show the mean shape of the structures.

Figure 4 shows the XRD patterns for the CuO-NPs synthesized from copper chloride and copper acetate. Principal peaks were located at 32.4°, 35.6°, 38.6°, 48.7°, 53.5°, 58°, 61.5°, 66.4°, 68°, 72.4° and 75.4° matching with those of CuO (crystallographic structure database COD: CuO #7,212,242) and were supported by analyzing the breadths of diffraction and spectral lines using the methods of Williamson-Hall and Langford (Figure S2) and the Rietveld refinement (anisotropic, isotropic and distribution) in whole-pattern fitting, WPF. The XRD spectra of the CuO-np and CuO-ns showed a pure monocrystalline phase of CuO with no impurities of Cu(OH)_2_ or Cu_2_O.

The size results obtained by TEM were in good agreement with data from the microstructural image analysis by XRD (insert in Figure 4). The average real size from XRD data was estimated using Equation 1, which is based on the Rietveld structure refinement in which simple analytical functions (Gaussian, Lorentzian, Pseudo-Voigt, Pearson VII) and empirical profile shape functions are used together with Scherrer’s formula [23]:

(1) $$\overset{ˉ}{D}=(4/3)\langle D\rangle_{V}$$

In this equation $\langle D\rangle_{V}$ is the weighted crystal apparent size in volume. Also, the average size was estimated using the simple Scherrer equation [24] from the broadening of XRD reflections (Equation 2):

(2) $$\bar{D}=\frac{57.3\kappa \times \lambda }{\beta \cos\theta }$$

where *D* = mean diameter, *k* = shape factor, *λ* = wavelength, *β* = the full-width at half maximum, and *θ* = Bragg angle for studied ring. The factor 57.3 is used for conversion of *β* from degree to radians. Results from both methods were in good agreement (Table S1). Besides, there is a good agreement of data based on the Scherrer formula with those by TEM as particle sizes were less than 60 nm [25].

### 3.3. EDXMA and XPS analysis

The elemental analysis of the copper nanoparticles was determined by Energy Dispersive X-Ray Microanalysis (EDXMA). Semi-quantitative analysis estimated about 40–51 atomic % Cu and 45–48 atomic % O contents in both, pristine CuO-ns and CuO-np, composition close to the theoretical one. Functional CuO-np contained ca. 0.7–0.8 atomic % of Si, while C detected in CuO-np-C_18_ was about (4 ± 2) atomic %. For functional CuO-ns the EDXMA analysis of C and Si was not conclusive probably due to their low concentration, insufficient for signal averaging. This fact corroborated our hypothesis of a low functionalization yield as already described in the FTIR section.

XPS analysis for CuO-np-C_18_ revealed a surface composition of 53% O, 32% C and 16% Cu. High-resolution spectra showed an intense (62% of the total area) photoelectron peak Cu2p_3/2_ at 933.4 eV and two weaker peaks at 942.8 eV (9% area) and 940.7 eV (28% area), characteristic shake-up satellite lines consistent with those described for crystalline CuO [26]. The XPS spectra also revealed the main O1s peak (78% total O) at 529.7 eV in agreement with O^2-^ in CuO the O1s (22%) at 531.8 eV may be a signature of adsorbed oxygen on the CuO surface [27]. Regarding the C1s band, most of carbon (85%) appears at 284.6 eV consistent with C–C/C-H bonds in alkyl chains [28]. The low contribution (15%) from C1s XPS peaks at higher binding energy at 287.8 eV could be assigned to C = O type bonds [29], probably due to adventitious carbon in the vacuum chamber or build up at the sample surface during spectra acquisition (detection depth of XPS is usually less than 10 nm).

### 3.4. TGA and SDTA analysis

**Figure 5.**
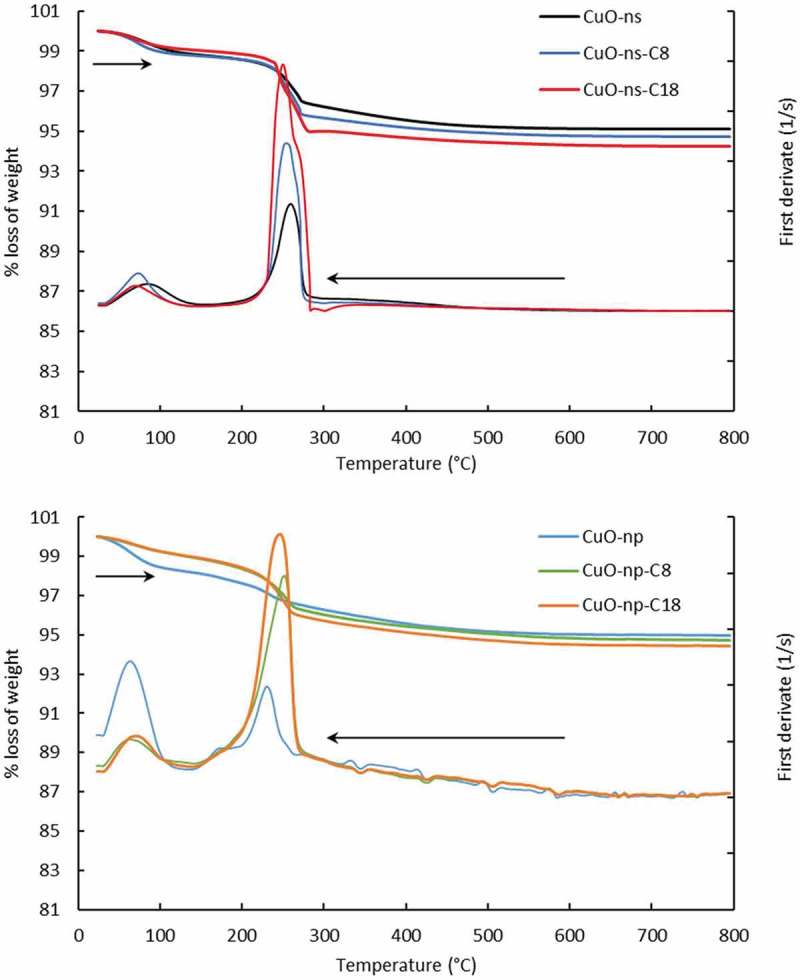
TGA and SDTA for CuO nanoparticles.

TGA and SDTA curves showed that the loss weight of nanoparticles took place in two steps (Figure 5). In the first one, below 100°C, a very slight weight loss about 1.1% and 1.5% for pristine CuO-ns and CuO-np, respectively, which corresponded to the removal of physically adsorbed water. The slightly higher water loss of CuO-np respect to CuO-ns could be explained by considering the higher surface area per mass unit of the first. Also, below 100°C, a low weight loss of about 0.9 ± 0.1% was observed for the alkylated C_8_- and C_18_- CuO-ns and CuO-np, due to their higher hydrophobic character.

In the second step, pristine and functional CuO-ns showed loss of weight in the temperature range of 193–293°C while raw and functional CuO-np in the range 185–273°C. Deconvolution of SDTA bands (Figure S4) showed that raw CuO-np presented a unique peak at ca. 229°C assigned to condensation of surface hydroxyl groups, while functional CuO-np presented a peak at ca. 219°C (with a contribution of 25% weight loss) and an additional peak (see Table 1) ascribed to thermal decomposition of chemically bonded alkyl chains (with a contribution of 75%).

**Table 1.** Fitting of SDTA second band from Figure 5.

CuO nanoparticles	Peak 1 (°C) -OH condensation	Peak 2 (°C) Alkyl chain decomposition	Peak 3 (°C) Acetate loss	Coverage degree mmol alkyl/µmol CuO NP
CuO-np	229	–	–	–
CuO-np-C_8_	219	249	–	0.46
CuO-np-C_18_	218	246	–	0.27
CuO-ns	249	–	260	–
CuO-ns-C_8_	236	253	266	0.23
CuO-ns-C_18_	242	253	272	0.21

On the other hand, deconvolution of SDTA bands for CuO-ns, showed that raw CuO-ns had two contributions, one at 249°C due to condensation of hydroxyl groups and other at 260°C assigned to loss of acetate precursors. Besides these two contributions with a contribution of 50% weight loss, functional CuO-ns presented an additional third peak at 253°C (Table 1) assigned to thermal decomposition of bonded alkyl chains.

The hydrophobic grafting introduced by alkylation was calculated (Table S2) as the ratio of the amount of alkyl groups per nanoparticle (mmol alkyl/µmol CuO nanoparticle), and it was estimated dividing the weight loss due to the thermal decomposition of chemically bonded alkyl chains by the weight of bare nanoparticles after calcination at 800°C. Results, summarized in Table 1, showed that the coverage degree of the CuO-ns was similar whatever the length of the bonded alkyl chain. On the other hand, as seen in Table 1 the coverage degree of CuO-ns was lower than that observed for CuO-np. This fact may be explained by the impact of steric hindrance due to the presence of acetate groups on the CuO-ns.

For functional CuO-np an increase in the coverage from 0.27 to 0.46 was accompanied by a decrease in the chain length from C_18_ to C_8_, respectively. These results stand in contradiction to reports stating that a more dense packing is observed for longer alkyl chains (C_18_ compared to C_8_) as a result of hydrophobic interactions between alkyl chains [30]. Although we do not exactly know the structure of the functional CuO-np surface, we suppose that this fact may be explained considering that the coverage of CuO-np was not isotropic due to not only the alkyl chain length but also to the nanoparticle shape. In fact, several nanoparticle shape intertwining factors should be considered for CuO-np: i) the different surface area of long axis and that of facets at the two ends of the nanoparticles [31], ii) the most active planes of CuO nanoparticles are {001} due to their high surface energy, thus providing more reactive points for functionalization [32,33] and iii) the curved CuO-np surface ends which may affect the kinetics of functionalization [34]. All these factors may be responsible for different kinetics and chemical interactions of short C_8_ and longer C_18_ alkyl chains to bind at distinct surface sites on the same type of CuO-np.

### 3.5. Optical absorption spectroscopy characterization

**Figure 6.**
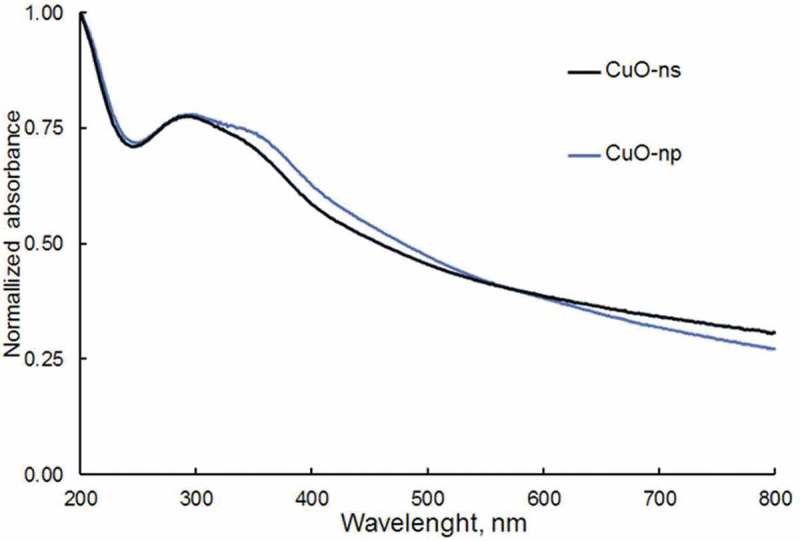
UV-Vis spectra for CuO nanoparticles in aqueous suspension.

The UV-Vis spectrophotometry is a very useful spectroscopic method to determine the energy gap and the optical properties of nanoparticles. The UV-Vis spectra of CuO nanoparticles in aqueous suspension are shown in Figure 6. CuO-ns and CuO-np, exhibit a maximum absorption peak at about 290 nm and a less intense at 380 nm, both bands due to the collective oscillation of the free conduction band electrons which are excited by the incident electromagnetic radiation (surface plasmon absorption, SPA). The SPA band at 380 nm indicates the formation of CuO nanoparticles [35] and assigned to the recombination of electrons in the conduction band to the holes in the valence band. When the copper nanoparticles are smaller than the Bohr excitonic radius the electronic and optical properties of nanoparticles are size dependent. In fact, the decrease in size of the nanoparticles enhances the quantum confinement effect that produces a spectral blue-shift [36]. Thus, the band at 290 nm can then be ascribed to the population of CuO-ns with sizes ranging 8–10 nm and to CuO-np of size 6–18 nm (Figure 3), smaller than the Bohr radius of CuO between 6.6 and 28.7 nm [37].

From the analysis of these spectra, the energy of the forbidden band (E_g_) for CuO-ns and CuO-np was estimated using the Tauc’s law for direct transitions [38]:

(3) $$(\alpha h\;v{)}^{2}=k(hv-E_{g})$$

where α is the absorption coefficient, hν the energy of the incoming light and k is a constant. Then, E_g_ can be estimated from the graphical representation of *(ahν)^2^* vs *hν* by extrapolating the straight line to (*ahν*)^2^ = 0. The obtained values of E_g_ are recorded in Table 2. E_g_ values obtained for both, CuO-ns (2.62 ± 0.01 eV) and CuO-np (2.47 ± 0.01 eV), were much higher than those reported for bulk CuO (1.5 eV) [39] but consistent with the values obtained for CuO nanoparticles by other researchers (2.36 eV, 2.03 eV) [40,41]

**Table 2.** Bandgap values of CuO nanoparticles.

Nanoparticles	$E_{g,suspension}^{Abs}$, eV	Excitation $\lambda_{g,solid}^{PL}$, nm	$E_{g,solid}^{PL}$, eV
CuO-ns	2.62 ± 0.01	266	2.62 ± 0.06
	4.91 ± 0.05		3.4 ± 0.1
CuO-np	2.47 ± 0.01	264	2.46 ± 0.05
	……		2.6 ± 0.1
	4.82 ± 0.06		3.4 ± 0.1

**Figure 7.**
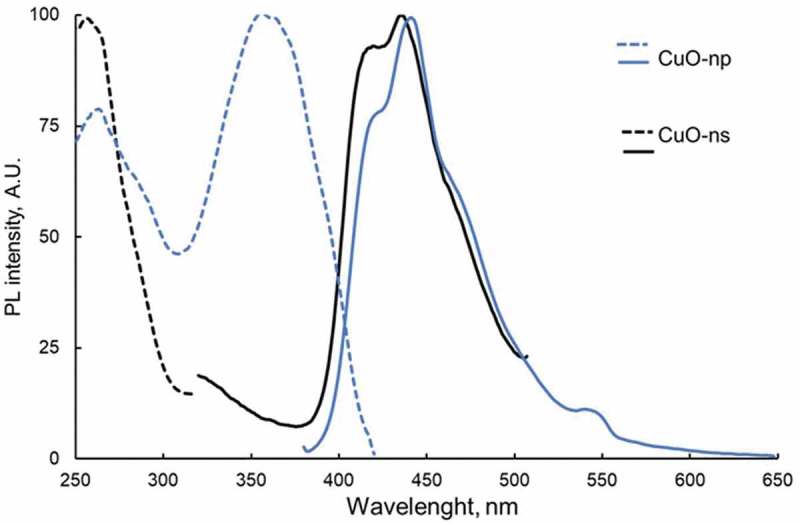
Photoluminescence spectra for dry CuO-ns and CuO-np samples (–) Excitation and (─) emission spectra.

The band gap values of dry CuO-ns and CuO-np were also estimated by photoluminescence (PL) as for CuO nanoparticles in suspension PL was not observed due to the high quenching effect of the solvent. The PL spectra of the nanoparticles are shown in Figure 7. CuO-ns exhibited a single excitation maximum at 264 nm and two luminescence emission maxima located at 417 and 437 and a shoulder at 437 nm. For CuO-np, two excitation bands at 264 and 360 nm were observed. Whichever the excitation wavelength, Cu-O np showed two PL bands at 424 nm and 442 nm and a weak shoulder at 469 nm. Furthermore, exciting at 264 nm showed a new low PL peak at 315 nm whereas a low-intensity peak at 548 nm appears when exciting at 360 nm. The PL bands at 424 nm and 442 nm of CuO-np are red-shifted (ca. 7 nm) compared to those of CuO-ns while the shoulder band of CuO-np is 32 nm red-shifted with respect to CuO-ns.

The excitation of semiconductor nanoparticles takes place when hν_exc_ ≥ E_conduction_ – E_valence_. So, there is a minimum energy, $E_{g}^{PL}$, required to promote an electron from the valence band to the conduction band. E_g_ can be estimated from Tauc’s law for direct transitions considering the intensity of PL proportional to the absorption coefficient α [42]. Therefore, Equation 1 can be transformed into Equation 4:

(4) $${\left(Fh\nu \right)}^{2}=k^{\prime }\left(hv-E_{g}^{PL}\right)$$

The $E_{g}^{PL}$ can be then estimated from the graphical representation of *(Fhν)^2^* vs *hν*. The obtained values for $E_{g}^{PL}$are summarized in Table 2.

There is a high concordance among the obtained values regarding the minimum energy difference between the valence band and the conduction band, as expected. Neither the chemical environment (solid, solution) nor the calculation methodology seems to influence significantly. Nevertheless, the most energetic transitions whose levels are more sensitive to the environment are indeed affected, appearing a blue-shift as a consequence of the destabilization of the energetic levels when the nanoparticles are in suspension.

### 3.6. Industrial applications

#### 3.6.1. Lubricant additives

Different suspensions of CuO-ns and CuO-np in base oil were prepared and tested in order to study the effect of the nanoparticle shape as well as the influence of the functionalization on the tribological properties of the oil. The effect of the shape of the nanoparticles was estimated by comparing the coefficient of friction (CoF) and the wear volume (W_D_) for suspensions of either raw CuO-ns or raw CuO-np in base oil with those of the neat base oil. The influence of the nanoparticles functionalization was carried out by comparing the CoF and W_D_ of CuO-np-C_8_ and CuO-np-C_18_ suspensions with those of the neat base oil.

**Figure 8.**
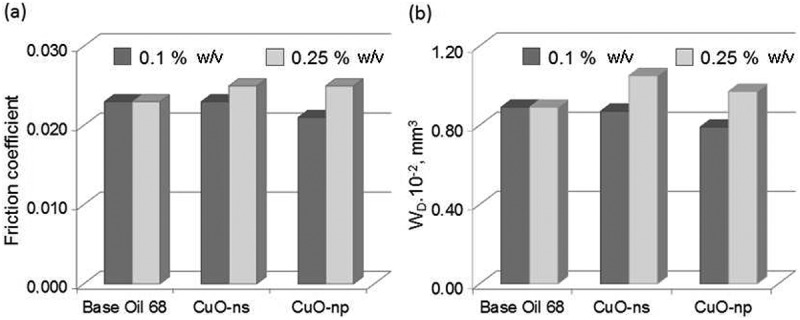
Effect of nanoparticle shapes on tribological properties at 0.1% and 0.25% w/v in BO68. (a) Friction coefficient and (b) wear.

Figure 8(a) clearly shows that a 0.1% w/v (weight/volume) CuO-np oil suspension improved the global lubrication performance, reducing both CoF and W_D_ (about 10%) compared to the neat base oil. This behavior can be attributed to a mending effect (protective layer formation) between the contacting bodies of CuO-np. It is worth mentioning that CuO-np lubricity properties worsened at high concentration (0.25%CuO-np oil suspension). This fact could be attributed to the aggregation of CuO-np between contacting surfaces which, under pressure, may increase the friction. Unlike CuO-np, performance of CuO-ns suspensions did not improve lubricity at any of the concentrations assayed.

**Figure 9.**
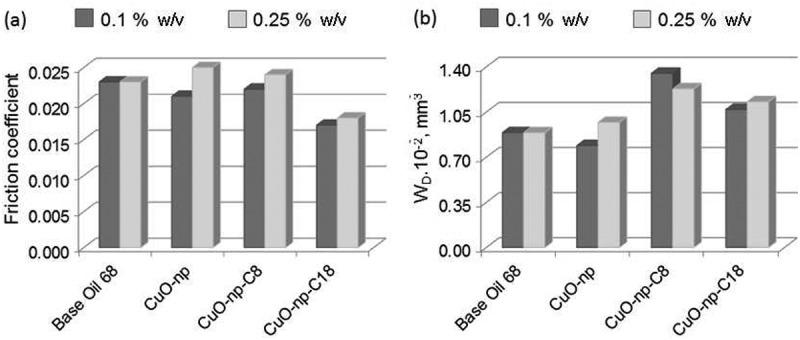
Effect of nanoparticle functionalization on tribological properties at 0.1% and 0.25% w/v in BO68. (a) Friction coefficient and (b) wear.

**Figure 10.**
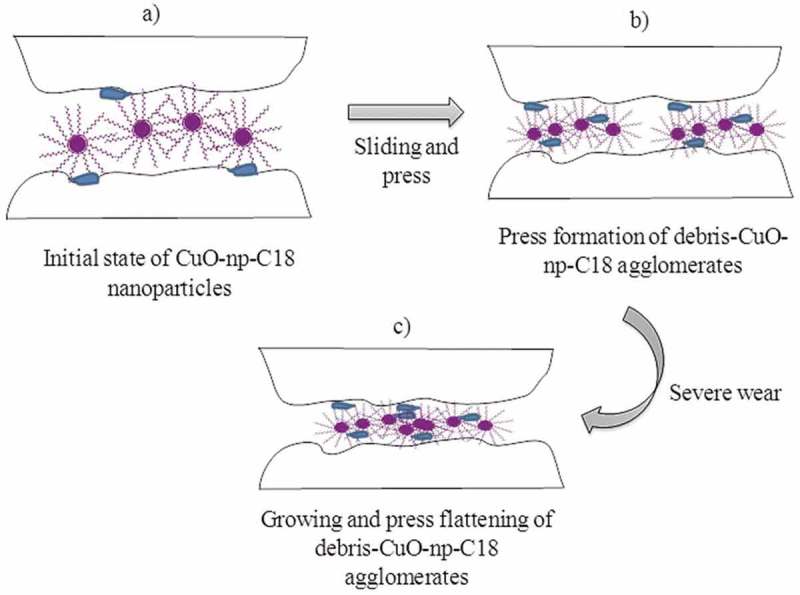
Proposed mechanism for the lubricant effect of nanoparticles.

The C_18_ functionalization of CuO-np (CuO-np-C_18_ in Figure 9(a)) improved the friction by reducing the CoF (ca. 26%) when compared to the base oil at both concentrations, 0.1% and 0.25% w/v. The alkyl chains not only stabilized the dispersion, but also acted as an additional soft protecting layer which minimized the hard nanoparticle interior contact with the rolling surfaces. Besides, as can be seen in Figure 9(a), the CoF difference observed for CuO-np-C_18_ at 0.1% and 0.25% w/v, is lower than in the case of CuO-ns-C_8_ nanoparticles. The reason behind may be that CoF reached a minimum when CuO-np-C_18_ concentration was 0.1% but CoF slightly increased when the concentration was higher, which probably resulted from aggregation of CuO-np-C_18_ in excessively high concentrations (in this case, more than twice). On the other hand, studies on the wear scar using five different chrome-steel disks revealed that the wear volume was larger for CuO-np-C_18_ oil suspensions, whatever the concentration used (Figures 8(b) and 9(b)). A plausible explanation could be that hard debris generated during sliding may be entrapped through mechanical mixing into the hydrophobic C_18_ surface of CuO-np-C_18_. At the same time, under high load conditions, these mixed debris-CuO-np-C_18_ may give to the formation of agglomerates during the assay. Upon agglomerates growing, they may become plastic deformed and sandwiched between the sliding surfaces. The dragging of these bigger agglomerated could create a bigger scar compared to the neat CuO-np, but without losing the ‘bearing effect’ (due to their hydrophobic soft surface) which reduced CoF. An illustration of the proposed mechanism is shown in Figure 10.

It was observed that a low concentration of CuO-np-C_18_ of 0.05% w/v reduced the wear, since the agglomeration probability also decreased. Simultaneously, the ‘bearing-effect’ also decrease, being the CoF 12% worse than that of the sample assayed with 0.1% w/v concentration. We can conclude that among the nanoparticles described this work, in comparison with the base oil, CuO-np-C_18_ offered potential as additives for nanolubrication due to the minimum friction coefficient, while CuO-np could be used as anti-wear additives due to the maximum wear reduction.

#### 3.6.2. Nanoparticles as dye-adsorption removers

Congo Red (CR) a water persistent benzidine-based azo dye (Figure S1), was used in this application as a model compound. CR, highly soluble in aqueous media, has been reported to be toxic and suspected carcinogen and mutagenic to many organisms, even for humans [40]. This dye is mainly used in the paper and textile industries due to its coloring properties and its presence in water bodies is due to industrial wastewater discharges without proper treatment.

Congo Red has been classified as a persistent pollutant, resistant to the biological degradation, due to its complex stable aromatic structure. However, it can be catalytically photo-decomposed [43]. Based on the fact that some metal oxide nanoparticles have been used as an efficient catalyst for degradation of hazardous dyes [44], a kinetic study of adsorption and degradation of Congo Red from environmental samples was performed in order to gain knowledge about how shape and surface modification of CuO nanoparticles may affect these processes.

**Figure 11.**
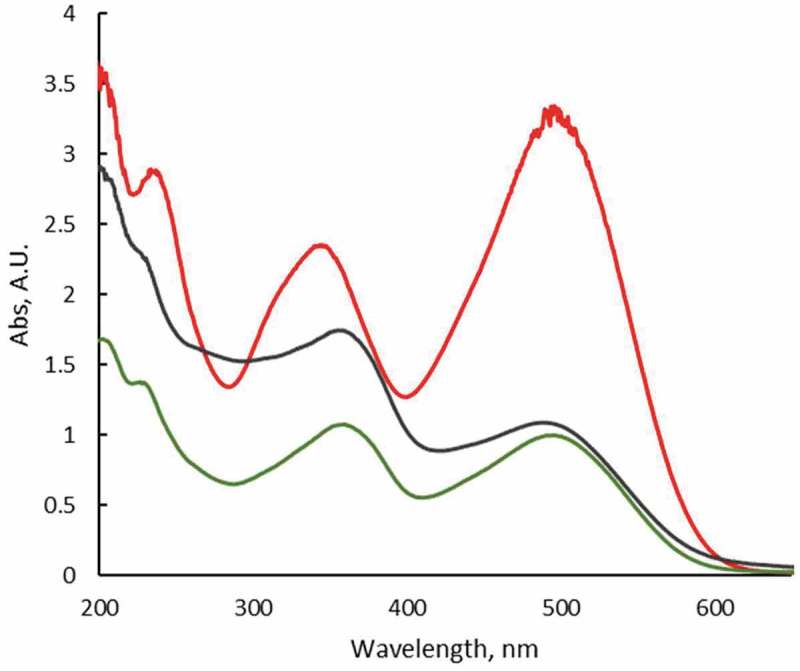
UV-Vis spectra of aqueous Congo Red solution (red line) and in contact with CuO-np (green line) and CuO-np-C_18_ (black line) in the absence of light. [CR] = 10^−3^ M.

The adsorption and degradation studies were monitored by UV-Vis spectrophotometry at room temperature following the changes in the absorption spectrum of Congo Red in aqueous solutions (Figure 11). As can be seen, the dye exhibits three bands centered at 240 and 350 (π-π* transitions in benzene and naphthalene rings, respectively) and 498 nm (n-π* transition of lone pair in the azo group). In contact with CuO-np and CuO-np-C_18_, under dark conditions (no photocatalytic effects) the UV-Vis spectra of the supernatant of these suspensions after 1.5 days, revealed no changes in the absorbance ratio of the dye main bands. This fact suggested that the chemical structure of the dye was not modified. However, there was an evident decrease in the absorbance, indicating a reduction in the concentration of Congo Red in the supernatant (ca. 65–70%) probably due to the adsorption of the dye onto the nanoparticles surface.

**Figure 12.**
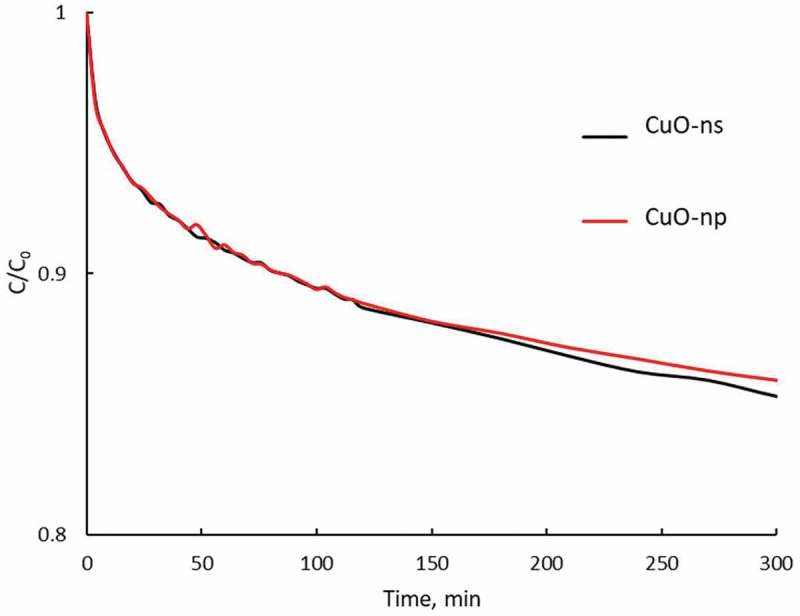
Evolution of Congo Red concentration in solution versus contact time with raw CuO in non-radiated conditions: 0.075 μM CuO-ns (black line) and 0.027 μM CuO-np (red line). Initial Congo Red concentration: 2.5 × 10^−4^ M.

The influence of nanoparticles shape on the adsorption phenomenon was evaluated by comparing the reduction in the absorbance of the supernatant of two solutions (2.5 · 10^−4^ M CR), one containing 7.5 · 10^−8^ M CuO-ns and the other containing 2.7 · 10^−8^ M CuO-np. With this aim, we estimated that suspensions of 7.5 · 10^−8^ M CuO-ns and 2.7 · 10^−8^ M CuO-np have a comparable nanoparticle surface. The surface of the nanoparticles was calculated as the surface of a 10 nm diameter sphere for CuO-ns while for CuO-np 22 nm-length and 6 nm-wide dimensions were used. The suspensions were kept in darkness for 10 h to avoid photocatalytic degradation of the dye. Figure 12 (time limited to 5 h) shows the evolution of the dye concentration remaining in solution versus time and as can be observed, the influence of CuO nanoparticles morphology on the adsorption process was marginal.

Adsorption kinetics was analyzed using pseudo-first order, pseudo-second order and intra-particle diffusion models [45]. According to the co-relation coefficient, R, between the experimental data and the values predicted by the different models, the best fit for the adsorption curves corresponded to a pseudo-second-order kinetics, according to equation 5:

(5) $$\frac{t}{C_{t}}=\frac{1}{K\cdot {C_{e}}^{2}}+\frac{t}{C_{e}}$$

where *C_t_* and *C_e_* represent the adsorbed dye concentration at a time *t* and the concentration in the equilibrium, respectively, *K* is the kinetic constant (data included in Table 3 for each Congo Red-nanoparticle system). *K* are not statistic significantly different for CuO-ns and CuO-np solutions due to their similar surface area; however, the CuO-np seemed to be more effective for adsorbing material if we take into consideration that a lower CuO-np concentration was needed to retain the same dye percentage as a higher CuO-ns concentration.

**Table 3.** Kinetic parameters for the adsorption process in darkness, fitted with a pseudo-second-order model.

		C_e_ µmol L^−1^	*K* L mol^−1^ min^−1^	*R*	Adsorbed dye %
Raw nanoparticles	CuO-ns	63	247	0.993	25.2
	CuO-np	65	273	0.991	26.0
Functional nanoparticles	CuO-np-C_8_	11	1024	0.994	4.4
	CuO-np-C_18_	8	2584	0.993	3.2

As CuO-np offered potential for Congo Red adsorption, similar experiments were then performed using the functional CuO-np nanoparticles and results for pseudo-second-order kinetics are included in Table 3. In comparison with raw CuO-np, the functional ones greatly affected the adsorption kinetics: the constant *K* increased about 4 and 9 times for CuO-np-C_8_ and CuO-np-C_18_, respectively. From data in Table 3, although the adsorption kinetics increases with the length of the alkyl chain, the adsorption efficiency of Congo Red was about 6 and 8 times higher for CuO-np than for CuO-np-C_8_ and CuO-np-C_18_, respectively.

We hypothesized that adsorption of Congo Red by CuO-np is due mainly to hydrogen bonding: the amine groups of Congo Red can form strong hydrogen bonds with the hydroxyl groups on CuO-np. However, a small amount of Congo Red could be adsorbed due to the exposition of charged sulfonic groups but in a limited extent due to steric hindrance among the long surface hydrocarbon chains of functional CuO-np-C_8_ and CuOnp-C_18_.

It is, therefore, expectable that raw CuO-np are affective as pre-concentration systems and as dye-removers. Likewise, long-chain alkyl functionalized nanoparticles should be more effective for the extraction of chemicals with high hydrophobic nature.

##### 3.6.2.1. Photodegradation study of congo red

**Figure 13.**
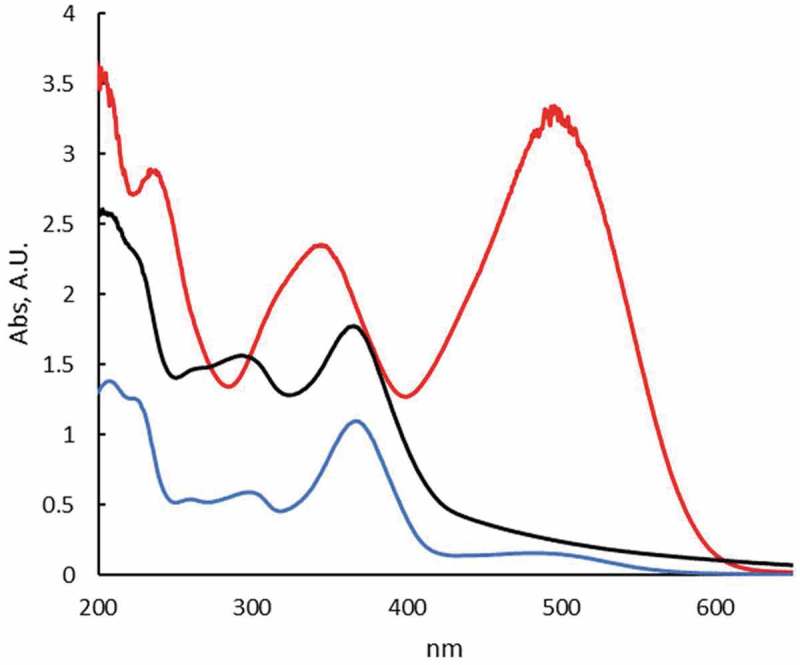
UV-Vis spectra of aqueous Congo Red solution (red line) and in contact with CuO-np (blue line) and CuO-np-C_18_ (black line) under sunlight. [CR] = 10^−3^ M. Irradiation time: 1.5 days.

In the absence of any nanoparticle, the degradation of CR under solar light proceeded at a very low pace (persistent dye), but as can be seen in Figure 13 an almost complete degradation was observed with CuO-np or with CuO-np-C_18_ under solar light after long time exposition. Figure 13 shows the vanishing of the band at 498 nm and the 20 nm blue-shift of the bands at 240 and 350, indicative of Congo Red degradation products formation. The evolution of the process was followed checking the absorbance at 498 nm of the nanoparticle suspension in a 1.0 · 10^−3^ M Congo Red solution. Equivalent suspensions kept in darkness during the same time interval of the study were used for comparison.

The final concentration of Congo Red in the sun-exposed suspensions either with functional CuO-np-C_18_ or raw CuO-np was below the detection limit of the used spectrophotometric method, which was interpreted as a 90–95% dye degradation. Similar results were obtained for raw CuO-ns and functional CuO-ns under illuminated conditions (data not shown). As tabulated in Table 4 our results compared favorably with other nanomaterials reported in the literature for the photochemical removal of Congo Red [20,46–49].

**Table 4.** CuO-np and CuO-np-C_18_ compared to the other materials for Congo Red photochemical removal.

Type of nanoparticles	Source of irradiation	Photochemical removal, %	Reference
ZnO	Sun light	85	[46]
CuO nano sheets	UV lamp	12	[14]
BiGdWO_6_	Sun light	90	[47]
TiO_2_	Hg lamp	100	[48]
Nanocomposite films	High pressure Hg lamp	85	[49]
CuO-np	Sun light	90	This work
CuO-np-C_18_	Sun light	95	This work

The mechanism for the catalytic action of CuO-np (or CuO-ns) and CuO-np-C_18_, should be different and we proposed that such difference can be found in the spatial restrictions forced by the long alkyl chains upon Congo Red, confining the dye in the hydrophobic shell and affecting the distance between the dye and the CuO surface itself.

**Figure 14.**
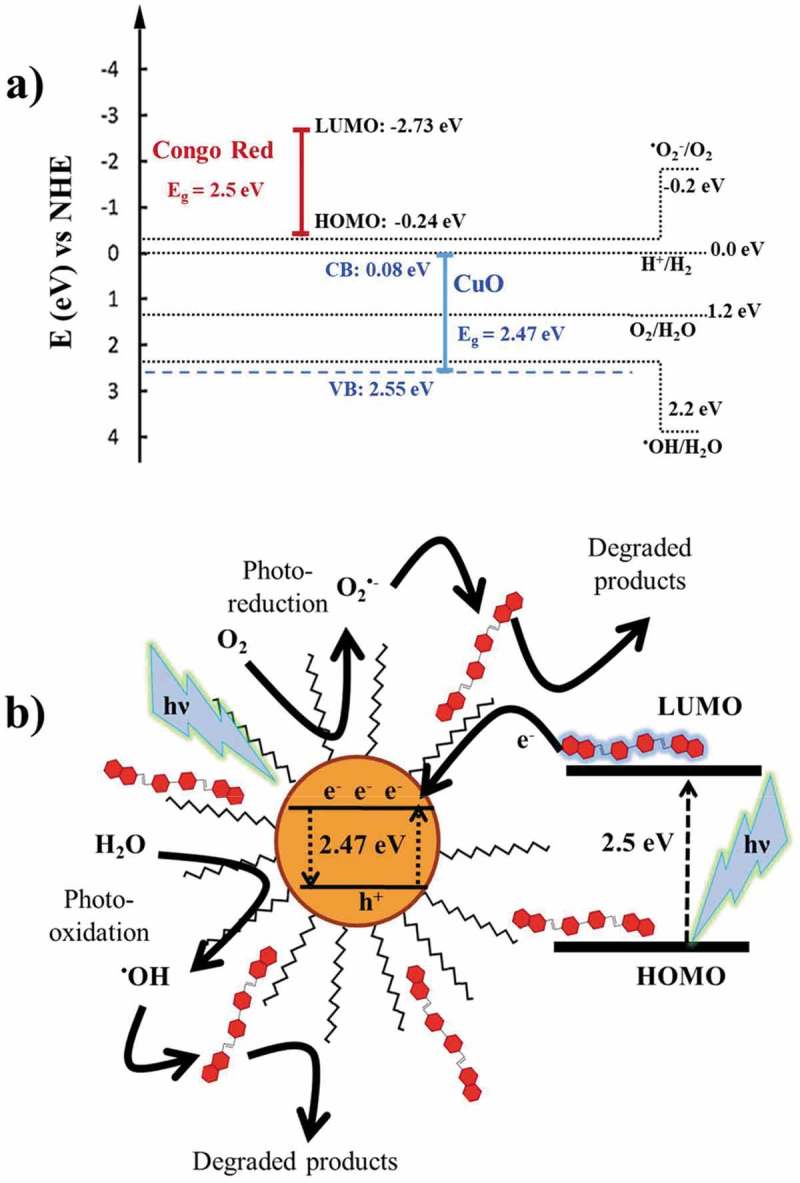
(a) Schematic diagram showing energy levels involved in the Congo Red photocatalytic degradation with CuO-np-C_18_; b) Graphical representation of radical species and charge transfer in the photocatalytic process.

CuO is a p-type semiconductor with a narrow bulk bandgap reported between 1.2 and 2.0 eV [50]. On the contrary, for CuO nanoparticles, as a result of the quantum confinement, larger band gaps have been also reported [16], which are in good agreement with our results. The red shift with decreasing particle size has been suggested to be due to intra-gap states as a result of surface defects [51]. When sunlight photons impinge on CuO NPs, some electrons (e^−^) are promoted from the valence band (VB) to the conduction band (CB), thus creating holes (h^+^) in the VB. These electron-hole pairs can recombine and release energy as photoluminescence but in the presence of surface materials (eg. O_2_, H_2_O, dyes, etc.) some photocatalytic processes could take place. For this last situation, optical key parameters of the CuO NPs must be considered: the band gap (E_g_) that corresponds to the difference between the energy levels of the top of the VB (E_VB_) and the bottom levels of the CB (E_CB_). E_CB_ and E_VB_ can be calculated using the expressions [52]:

(6) $$E_{CB}=\chi -E^{C}-(E_{g}/2)$$

(7) $$E_{VB}=E_{CB}+E_{g}$$

in which E_CB_ and E_VB_ are the potentials of the conduction and valence bands, respectively, E^C^ is the free energy corresponding to the hydrogen scale (~4.5 eV) [52], E_g_ and χ are the band gap and the electronegativity of CuO NPs, respectively. The χ is defined as the geometric mean of the absolute electronegativities of the Cu and O atoms. In our calculations, the electronegativity values for Cu and O were taken as 4.48 for Cu and 7.54 for O [53]. The E_CB_ and E_VB_ values for CuO-np-C_18_ are shown in Figure 14(a), in which the relative positions of CB and VB and redox potentials for the couples H_2_O/^•^OH (2.2 eV vs NHE) and O_2_/O_2_^•-^ (−0.18 eV vs NHE). According to these data, the CB of CuO-np-C_18_ is higher than the redox potential of the system O_2_/O_2_^•-^ and, consequently, the electrons in the CB could not reduce O_2_ to O_2_^•-^ radicals. However, contribution from extra electrons to the CB could overcome the energy difference between CB of CuO-np-C_18_ and the redox potential of O_2_/O_2_^•-^ so that a tunneling charge transfer could be a dominant path to populate the CB. The extra electrons to the CB of CuO-np-C_18_ can be provided by the excited CR, when illuminated under Vis-UV light which, at the same time, avoids recombination of electron-holes.

The energy gap (ΔE) of CR (2.5 eV, absorption maximum at 498 nm) may provide important information about the interaction with the CuO-np-C_18_ particles. The E_HOMO_ for CR has been reported at −4.26 eV (−0.24 eV vs NHE) [54]. It follows from our calculations that the energy of the LUMO for CR (ΔE = E_LUMO_− E_HOMO_) is −2.73 eV vs NHE, which is more negative than the CB of CuO-np-C_18_ and then, the excited CR electrons will most likely transfer to the CB of the nanoparticle. In this conjecture, we expect that this electron transfer could provide sufficient driving force the CuO surface reaction with dissolved O_2_ to produce O_2_^•-^ radicals.

On the other hand, the VB of CuO-np-C_18_ lies below the redox potential of the couple H_2_O/^•^OH what suggests that the holes generated during sunlight absorption by CuO-np-C_18_ could oxidize surface H_2_O to ^•^OH radicals. Both, O_2_^•-^ and ^•^OH are the primary initiators for the photocatalytic degradation of Congo Red. The proposed mechanism for Congo Red photocatalytic degradation on the CuO-np-C_18_ is shown in Figure 14(b).

The confinement of Congo Red within the surface alkyl chains not only allowed it to inject electrons into the CV of the CuO-np-C_18_ to enhance the photocatalytic activity of the nanoparticles, but also the alkyl chains protected the CuO-np core from corrosion which, in turn, makes CuO-np-C_18_ a good candidate for removal persistent dyes from water.

## Conclusions

We have successfully coated copper oxide nanoparticles with alkyl groups. The new materials have been fully characterized by different spectroscopic tools. These functional CuO nanoparticles were highly stable in oily suspensions compared to the naked CuO ones and their hydrophobic surface may protect them from corrosion. We have demonstrated the applicability of the new materials both in tribology and as dye removers. From a tribological point of view, it has been verified that functional alkyl-CuO nanoparticles as lubricant base oil additives reduced the coefficient of friction, while from an environmental viewpoint the use of both raw CuO and functional alkyl-CuO nanoparticles could be good candidates as dye removers (adsorption and/or photocatalysis) for treating wastewater having persistent dyes as Congo Red.

## Supplementary material

The supplemental data for this article can be accessed here
