# Supplementary material for: Alkyl-capped copper oxide nanospheres and nanoprolates for sustainability: water treatment and improved lubricating performance
Source: Sci Technol Adv Mater. 2019 Jun 26;20(1):657–72. doi: 10.1080/14686996.2019.1621683 (PMC6598526; doi:10.1080/14686996.2019.1621683)
Supplement: Supplemental Material [file TSTA_A_1621683_SM6700.docx]

**Supplementary information**

**Alkyl-capped copper oxide nanospheres and nanoprolates for sustainability: water treatment and improved lubricating performance**

Christian Chimeno Trinchet, Alfonso Fernández-González, Josefa Ángela García Calzón, Marta Elena Díaz García, Rosana Badía-Laíño

Faculty of Chemistry, Department of Physical and Analytical Chemistry,

University of Oviedo. Av. Julián Clavería, 8 33006-Oviedo, Spain

[rbadia@uniovi.es](mailto:rbadia@uniovi.es), medg@uniovi.es

**Figure S1.** Main compounds used in the surface nanoparticle modification synthesis and Congo Red structure


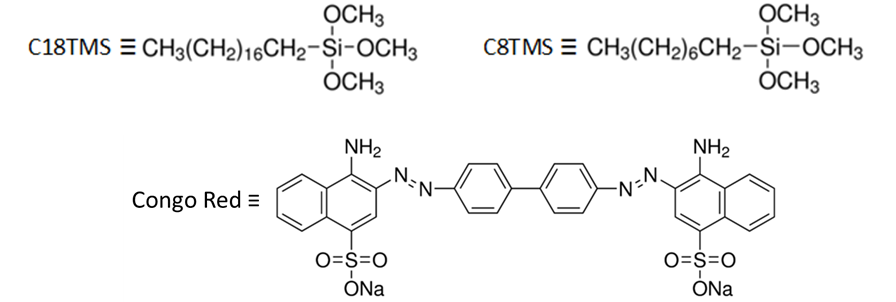


**Figure S2.** Linear and quadratic Williamson-Hall diagrams and Langford plot corresponding to the CuO-np sample

**
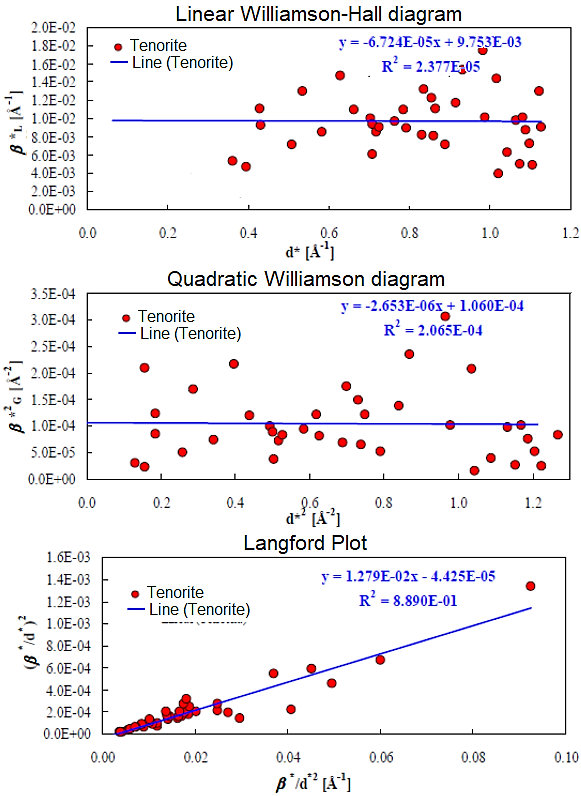
**

**Figure S3.** ATR-FTIR spectra for CuO nanospheroids (prolates) and silane coupling agents for surface nanoparticles modification.


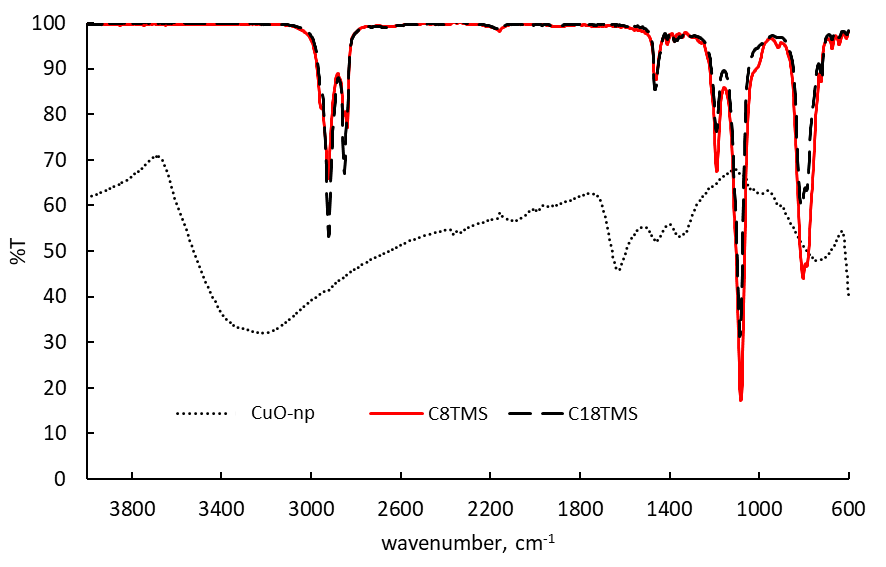


**Figure S4.** Deconvolution of SDTA bands (**⋅⋅⋅⋅⋅**) of synthetized nanomaterials assigned to: fitted band (**⎯**) condensation of surface hydroxyl groups (**⎯**), decomposition of chemically bonded alkyl chains (**⎯**) and acetate loss (**⎯**).

**
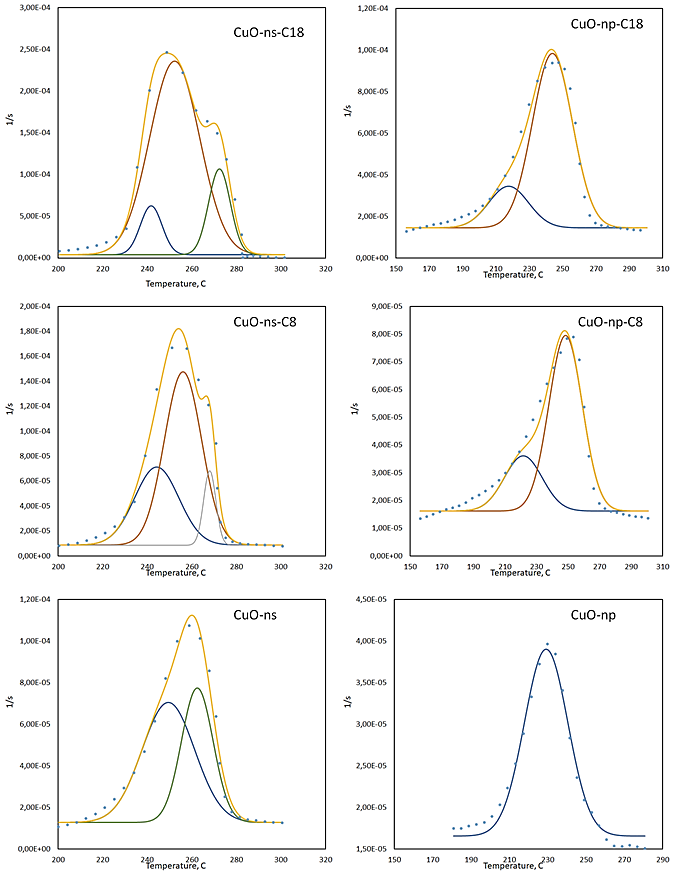
**

**Table S1.** Structural parameters derived from Rietveld analysis of XRD patterns for **CuO-ns** and **CuO-np** samples.

| **Sample** | **Microstructural Analysis** | | | |
| --- | --- | --- | --- | --- |
|  | **Rietveld refinement** | | **Debye-Scherrer** | **TEM** |
|  | **R_hkl_** [nm]  s [nm] | **R** [nm]  **σ** [nm] | **D_v_** [nm]  **σ** [nm] | **D (nm)**  **σ** [nm] |
| **CuO-ns** | 8.4  ±0.7 | 8.3  ±0.31 | 12.71  ±0.05 | 10.00  ±2.00 |
| **CuO-np** | 10.21  ±1.66 | 10.71  ±0.04 | 15.30  ±0.03 | Rod shaped  L = 22  D = 6 |

**Table S2.** Hydrophobic grafting introduced by alkylation

|  |  |  |  |  | **Alkyls** | | **Nanoparticles** | |  |
| --- | --- | --- | --- | --- | --- | --- | --- | --- | --- |
|  | **started weight, mg** | **mg CuO at 800 C** | **mg lossed** | **% alkyl by fitting SDTA bands** | **mg** | **mmols** | **number** | **μmols** | **mmol alkyl/µmol CuO-NP** |
| **CuO-ns** | 79,6120 | 75,7277 | 1,7506 | 0 | 0,0000 | 0,000 | 2,295E+16 | 0,0381 | 0,00 |
| **CuO-ns-C_8_** | 75,5990 | 71,5931 | 2,1318 | 43 | 0,9167 | 8,112E-03 | 2,169E+16 | 0,0360 | 0,23 |
| **CuO-ns-C_18_** | 85,2379 | 80,3122 | 3,2378 | 66 | 2,1370 | 8,447E-03 | 2,434E+16 | 0,0404 | 0,21 |
| **CuO-np** | 34,3187 | 32,5980 | 0,6094 | 0 | 0,0000 | 0,000 | 9,88E+15 | 0,0164 | 0,00 |
| **CuO-np-C_8_** | 33,5368 | 31,7765 | 0,9384 | 74 | 0,6944 | 6,145E-03 | 8,11E+15 | 0,0135 | 0,46 |
| **CuO-np-C_18_** | 39,2183 | 37,0351 | 1,3306 | 81 | 1,0778 | 4,260E-03 | 9,45E+15 | 0,0157 | 0,27 |
